# hMYH and hMTH1 cooperate for survival in mismatch repair defective T-cell acute lymphoblastic leukemia

**DOI:** 10.1038/oncsis.2016.72

**Published:** 2016-12-05

**Authors:** S Eshtad, Z Mavajian, S G Rudd, T Visnes, J Boström, M Altun, T Helleday

**Affiliations:** 1 Science for Life Laboratory, Division of Translational Medicine and Chemical Biology, Department of Medical Biochemistry and Biophysics, Karolinska Institutet, Stockholm, Sweden

## Abstract

hMTH1 is an 8-oxodGTPase that prevents mis-incorporation of free oxidized nucleotides into genomic DNA. Base excision and mismatch repair pathways also restrict the accumulation of oxidized lesions in DNA by removing the mis-inserted 8-oxo-7,8-dihydro-2'-deoxyguanosines (8-oxodGs). In this study, we aimed to investigate the interplay between hMYH DNA glycosylase and hMTH1 for cancer cell survival by using mismatch repair defective T-cell acute lymphoblastic leukemia (T-ALL) cells. To this end, *MYH* and *MTH1* were silenced individually or simultaneously using small hairpin RNAs. Increased sub-G1 population and apoptotic cells were observed upon concurrent depletion of both enzymes. Elevated cell death was consistent with cleaved caspase 3 accumulation in double knockdown cells. Importantly, overexpression of the nuclear isoform of hMYH could remove the G1 arrest and partially rescue the toxicity observed in hMTH1-depleted cells. In addition, expression profiles of human DNA glycosylases were generated using quantitative reverse transcriptase–PCR in *MTH1* and/or *MYH* knockdown cells. *NEIL1* DNA glycosylase, involved in repair of oxidized nucleosides, was found to be significantly downregulated as a cellular response to *MTH1*–*MYH* co-suppression. Overall, the results suggest that hMYH and hMTH1 functionally cooperate for effective repair and survival in mismatch repair defective T-ALL Jurkat A3 cells.

## Introduction

Genomic DNA is under constant attack by reactive oxygen species that occur naturally as by-products of aerobic metabolism. Increased production of reactive oxygen species is widely considered to be a consequence of malignant transformation in many cancer types,^1, 2^ and thus efficient repair of oxidized DNA lesions is considered critical for cancer cell survival.^3, 4^ Perhaps the most abundant and extensively studied oxidized DNA lesion is 8-oxo-7,8-dihydro-2'-deoxyguanosine (8-oxodG).^5^

Three major DNA repair pathways are thought to be responsible for preventing the accumulation of oxidized lesions in genomic DNA (Supplementary Figure S1). Oxidation can occur in the deoxyribonucleotide (dNTP) pool where the MutT homolog-1 (hMTH1) enzyme, encoded by the *NUDT1*/*MTH1* gene, hydrolyses 8-oxodGTP and other oxidized dNTPs into the monophosphate form.^6^ hMTH1 thus sanitizes the dNTP pool to avoid mis-incorporation of 8-oxodGTP into DNA during replication.^7, 8^ When 8-oxodG is already present in the DNA strand, replicative polymerases could incorporate either deoxycytidine triphosphate (dCTP) or deoxyadenosine triphosphate (dATP) opposite the lesion in the next round of replication, where 8-oxodG is in *anti* or *syn* conformation, respectively.^9, 10^ The 8-oxodG:C pairs are thought to be mainly detected and further processed by 8-oxoguanine DNA glycosylase (OGG1) that performs the first step of base excision repair (BER), removing the oxidized guanine paired with cytosine.^11^ The 8-oxodG:A mispairs are recognized by another DNA glycosylase called MutY homolog (hMYH, encoded by *MYH* gene) that excises the mis-incorporated dA in the nascent DNA strand. This provides the BER polymerases with an opportunity to correctly insert dCTP opposite 8-oxodG and thus create a substrate for OGG1.^12, 13^ On the other hand, the activity of hMYH on 8-oxodG:A where dA is in the template strand can potentially lead to T:A >>G:C mutations, and in this case, it has been hypothesized that the mismatch repair (MMR) pathway could contribute to the removal of the 8-oxodG in the nascent strand.^14, 15, 16^

In order to investigate the interplay between hMTH1- and hMYH-dependent BER, we utilized MMR defective T-cell acute lymphoblastic leukemia (T-ALL) cells.^17^ Malfunctioned MMR and the consequent microsatellite instability are highly frequent in T-ALL and thus simplify our study model.^18, 19, 20, 21^ In addition, RNA-sequencing analysis of over 900 human cancer cell lines revealed that T-ALL cells have the highest expression for both *MTH1* and *MYH*, making them an ideal model for this study^22^ (Supplementary Figure S2). To achieve simultaneous silencing of both genes, small hairpin RNA (shRNA) sequences from The RNA Consortium (TRC) library were cloned into doxycycline (Dox)-inducible vectors containing different fluorescent reports (green fluorescent protein (GFP) or RFP670), followed by lentiviral transduction of Jurkat A3 cells. Next, the top 15% double positive cells were sorted using fluorescence-activated cell sorting (FACS). The cells were cultured in the presence of Dox and samples were harvested after 96 h for downstream assays.

Here, we observed higher cell death following *MTH1* and *MYH* double knockdown (KD) compared with individual KD. Expression profiling of human DNA glycosylases revealed their responses to the lack of hMTH1 and hMYH enzymes. Taken together, our results indicated that hMTH1 and hMYH functionally cooperate for cell survival in T-ALL Jurkat A3 cells.

## Results

### Efficient individual and simultaneous KD of *MYH* and *MTH1*

**Figure 1:**
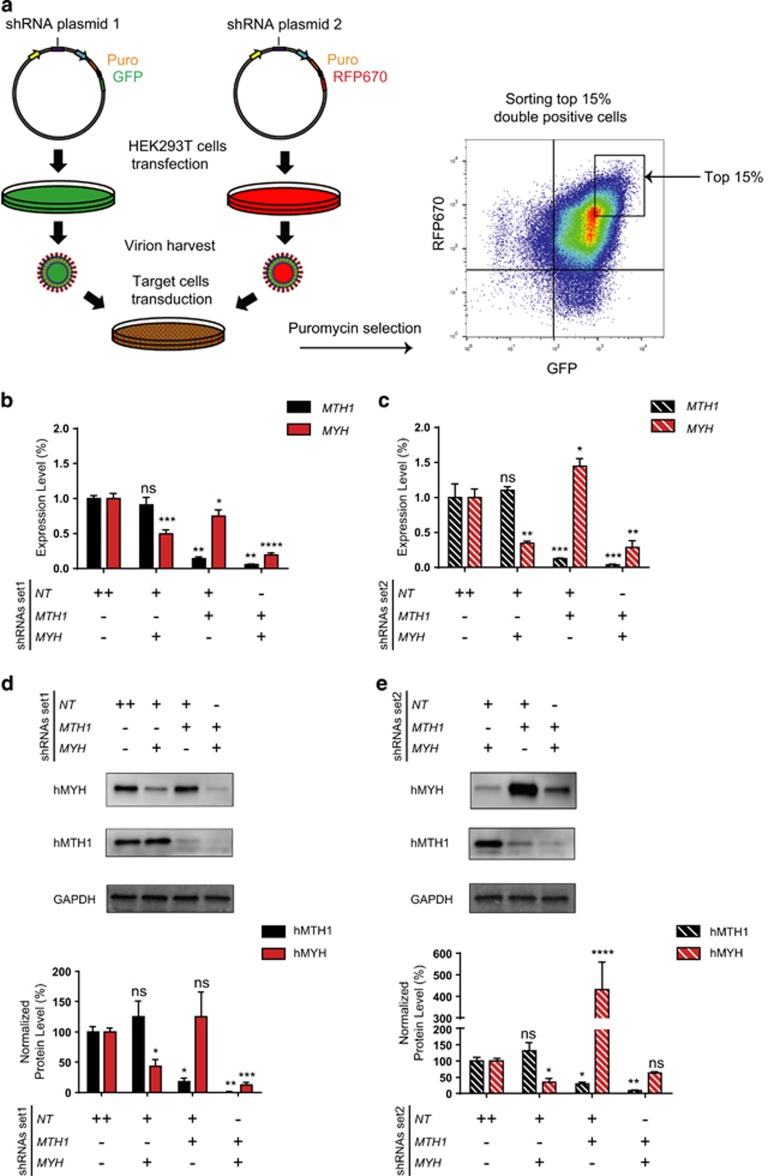
Simultaneous suppression of *MTH1* and *MYH* was efficiently achieved using a two-vector system. (**a**) Steps of cell line establishment are illustrated here. Top 15% of cells expressing GFP and RFP670 were sorted to improve the knockdown efficiency. Expression levels of *MYH* and *MTH1* were analyzed after 96 h of treatment with Dox in shRNA set1 (**b**) and shRNA set2 (**c**) using qRT–PCR analysis. Data were normalized to NT-shRNA expressing cells and presented as mean±s.e.m. from three independent experiments in triplicate. To investigate the knockdown efficiency at protein level, western blot analysis was performed after 96 h of Dox treatment of cells with shRNA set1 (**d**) and shRNA set2 (**e**). Data were double normalized with GAPDH and NT-shRNA control samples and expressed as percentage. The graphs represent mean±s.e.m. from three independent experiments. *P-*values were calculated using one-way analysis of variance (ANOVA). *P<0.05, **P<0.01, ***P<0.001, ****P⩽0.0001, NS, not significant.

To study the potential functional interactions between hMYH and hMTH1, two different shRNA sequences for each gene were cloned into separate inducible vectors containing either GFP or RFP670 fluorescent reporters. T-ALL Jurkat A3 cells were then co-transduced with lentiviral particles to establish stable cell lines concurrently expressing two different hairpins. Puromycin selected cells were subsequently sorted for the highest expression of both reporters (Figure 1a) and KD efficiency of each hairpin was analyzed using quantitative reverse transcriptase–PCR (qRT–PCR) (Figures 1b and c). Under these conditions, both *MYH* shRNAs were able to significantly suppress the expression of their target gene when co-expressed with a not-targeting hairpin. Similarly, *MTH1* was also significantly down regulated by its respective shRNAs. Furthermore, simultaneous silencing of both genes was successfully achieved when *MTH1* and *MYH* hairpins were used in combination. Interestingly, following 96 h of KD, *MTH1*-shRNA1 showed a suppressive effect on *MYH* expression (Figure 1b). In contrast, *MTH1*-shRNA2 resulted in a slight increase in *MYH* mRNA level (Figure 1c). We next validated hairpin activities at protein level using western blot analysis (Figures 1d and e), and obtained results comparable to the gene expression data. At both time-points, hMYH protein level was considerably reduced in cells expressing *MYH*-shRNAs separately or in combination with *MTH1*-shRNAs. Similar to qRT–PCR results, the hMTH1 protein KD was more efficient compared with hMYH, regardless of whether its corresponding shRNAs were used individually or co-expressed with *MYH* hairpins. Interestingly, in the cells containing single *MTH1*-shRNA2, a dramatically increased hMYH level was observed (Figure 1e). Possibly because of this effect, hMYH depletion level after 96 h was not statistically significant when the *MYH* hairpin was combined with *MTH1*-shRNA2. Thus, hMYH protein KD 96 h post treatment was more efficient when *MYH*-shRNA2 was used separately rather than in combination with *MTH1*-shRNA2. Collectively, these results demonstrate highly efficient individual and combinatorial KD of the both genes.

### hMYH and hMTH1 functionally collaborate to sustain cell survival in a T-ALL cell line

**Figure 2:**
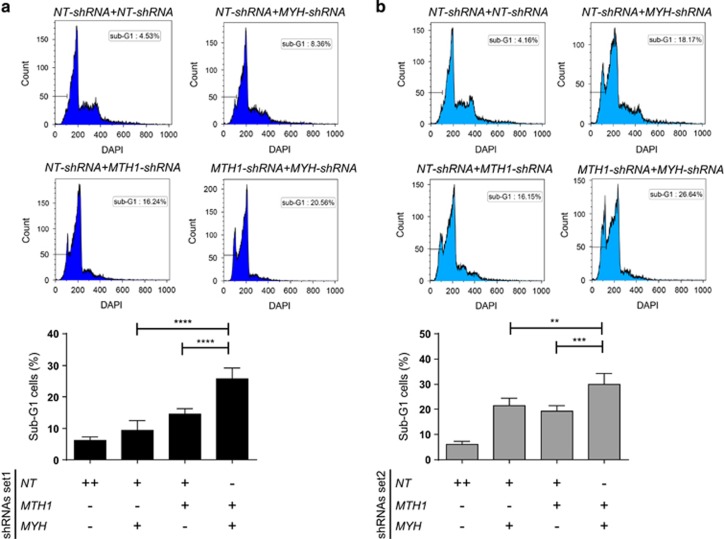
hMTH1–hMYH combined depletion significantly increased sub-G1 population. The percentage of sub-G1 population, indicative of cell death, was measured after 96 h of Dox treatment of cells with shRNA set1 (**a**) and shRNA set2 (**b**). One representative DAPI histogram is displayed for each condition. Quantified data are shown in bar charts representing mean±s.e.m. from three independent experiments in duplicate. *P-*values were calculated using one-way analysis of variance (ANOVA). **P<0.01, ***P<0.001, ****P⩽0.0001.

We first investigated the effect of *MTH1* and *MYH* double KD on survival of Jurkat A3 cells. On one hand, high DNA 8-oxodG caused by *MTH1* KD could lead to more putative DNA strand breaks mediated by hMYH, causing cell death. Therefore, based on this hypothesis, hMYH depletion would rescue the toxicity induced by the lack of hMTH1.^23^ On the other hand, hMYH hyperactivation could be a compensatory mechanism in the context of hMTH1 inhibition. Accordingly, a synergistic lethality would be expected as a consequence of combinatorial suppression of both enzymes. To test these hypotheses, we measured the percentage of sub-G1 cells upon Dox treatment in the cells with shRNA-set1 and shRNA-set2 at 96 h time-point. We observed concurrent KD of both proteins induced more cell death compared with individual depletions in both hairpin sets (Figure 2). These observations support the hypothesis that hMYH function is required for cell survival when hMTH1 activity is suppressed.

**Figure 3:**
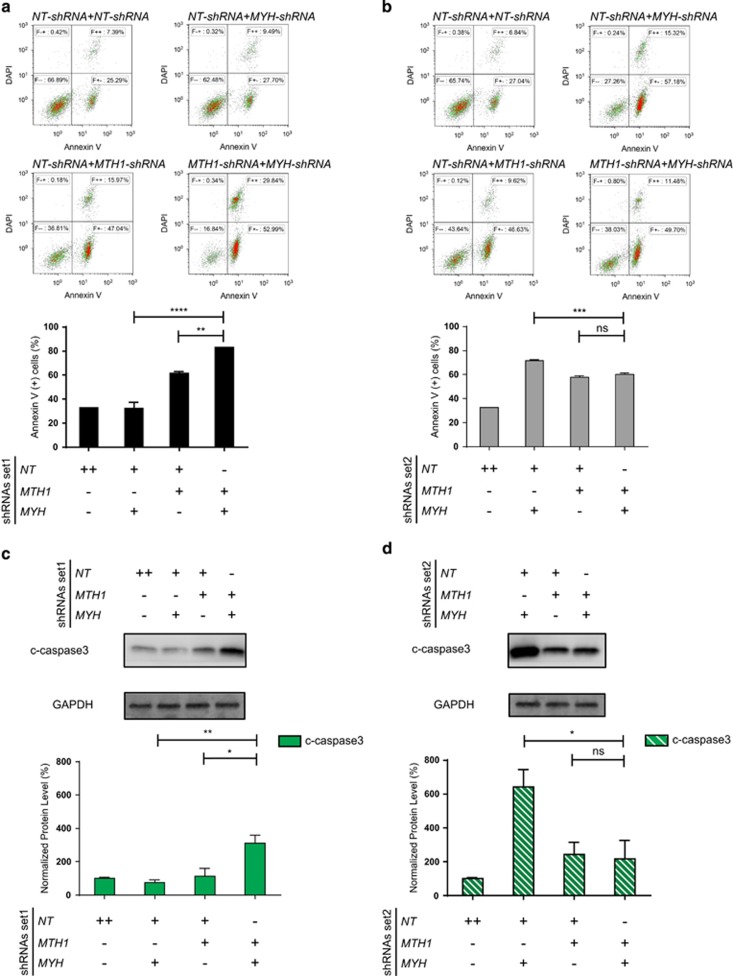
hMYH level dictates the apoptosis level in hMTH1-depleted cells. Apoptosis induction was 96 h post treatment with Dox by measuring Annexin V-positive population in the cells expressing shRNA set1 (**a**) and shRNA set2 (**b**). One representative DAPI versus Annexin V scatter plot is shown for each condition. Quantified data are illustrated in bar charts representing mean±s.e.m. from three independent experiments in duplicate. The same membranes in Figure 1 were used to measure the induction of apoptosis (cleaved (c)-caspase 3) after 96 h of Dox treatment in the cells expressing shRNA set1 (**c**) and shRNA set2 (**d**) The signals were quantified using Image Studio Lite (LI-COR), and double normalized with GAPDH and NT-shRNA control samples. One-way analysis of variance (ANOVA) was performed to calculate *P-*values. *P<0.05, **P<0.01, ***P<0.001, ****P⩽0.0001, NS, not significant.

To further understand how simultaneous KD of both genes influences survival in T-ALL cells, we studied induction of apoptosis using specific markers such as Annexin V and cleaved caspase 3 (Figure 3). We observed that the percentage of Annexin V-positive cells was higher in combined KD shRNA set1 compared with single KD of either genes (Figure 3a). Intriguingly, there was no significant difference in apoptosis level between the cells with combined KD and the ones expressing *MTH1*-shRNA2 (Figure 3b), and this could be owing to the increased hMYH level following *MTH1*-shRNA2 KD. As total cell death is also increased in the *MTH1/MYH*-shRNA2 KD, a more likely explanation is that the timing of apoptosis is such that the number of actively apoptotic cells at 96 h is lower. In addition, measurement of cleaved caspase 3 confirmed the results of Annexin V analysis (Figures 3c and d). Taken together, these results support the hypothesis that hMYH is required for cell survival in the absence of hMTH1.

### hMYH overexpression can rescue G1 arrest and cell death in *MTH1* KD cells

**Figure 4:**
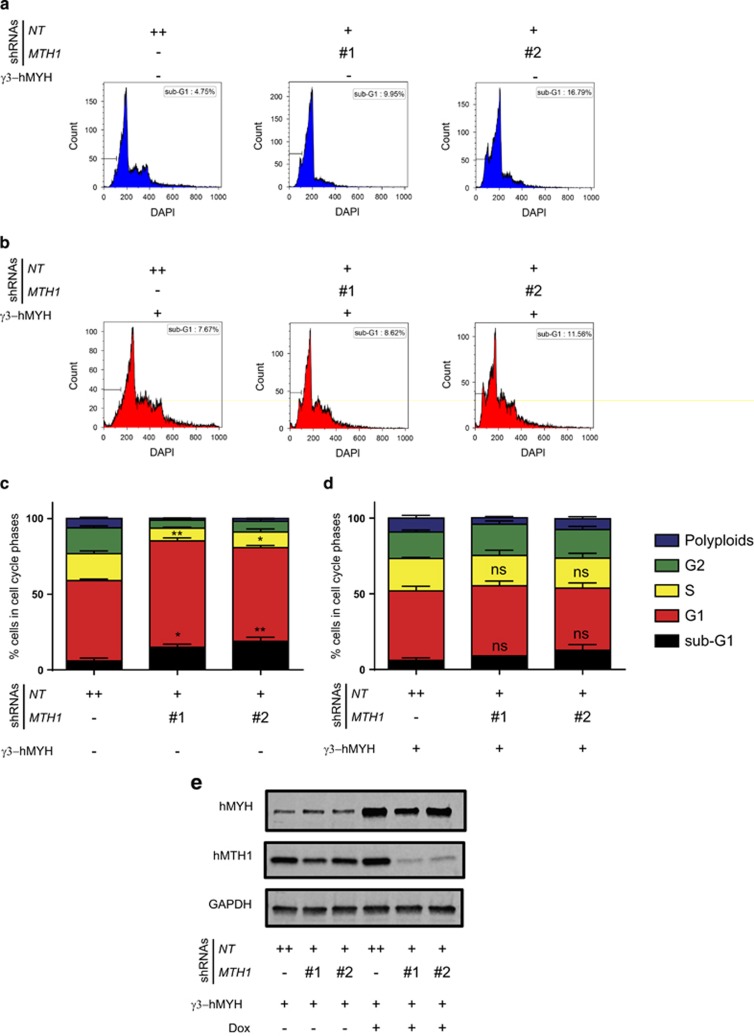
Cell death and G1 arrest in hMTH1-depleted cells were rescued by hMYH overexpression. DNA content of cells without (**a**) or with (**b**) γ3-hMYH overexpression at 96 h of Dox treatment is shown in histograms and their corresponding cell cycle distribution are displayed in stacked bar charts in (**c**, **d**). The γ3-hMYH overexpression was validated after 96 h of Dox treatment by western blotting. One representative membrane is displayed in **e**. The results are presented as mean±s.e.m. from at least two independent experiments in duplicate. *P-*values were calculated using one-way analysis of variance (ANOVA). *P<0.05, **P<0.01, NS, not significant.

To further test our hypothesis that hMYH is required for cell survival in the absence of hMTH1, we speculated that hMYH overexpression could rescue the phenotypes observed following hMTH1 depletion. To test this, we transduced the individual *MTH1*-shRNA cell lines with a Dox-inducible construct overexpressing a nuclear isoform of hMYH (γ3) and performed cell cycle analysis following 96 h of treatment with Dox (Figure 4). We observed that hMYH overexpression could remove the G1 block introduced by hMTH1 depletion. Furthermore, increased γ3-hMYH activity was shown to partially rescue the cell death mediated by *MTH1* suppression. These data further support that hMYH levels dictate the cell survival to hMTH1 depletion, and suggest a protective role for hMYH.

### *NEIL1* expression was suppressed upon combined depletion of hMYH and hMTH1

**Figure 5:**
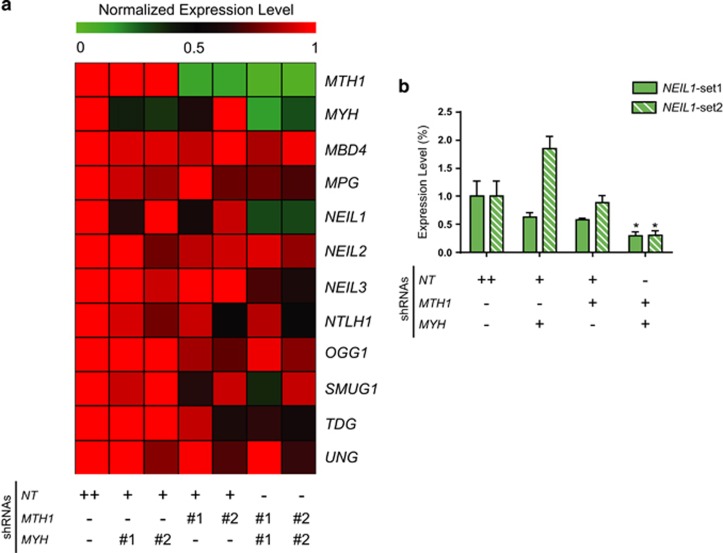
*NEIL1* was downregulated upon concurrent suppression of *MTH1* and *MYH.* (**a**) Expression of DNA glycosylases were analyzed after 96 h of Dox treatment using qRT–PCR and the results are shown in a heat-map diagram. (**b**) Detailed *NEIL1* expression levels are represented in a bar chart. Data are presented as mean±s.e.m. from three independent experiments in triplicate. *P-*values were obtained using one-way analysis of variance (ANOVA). *P<0.05.

*MTH1* and *MYH* double KD, in a MMR-defective background, would presumably lead to high levels of oxidized DNA lesions. Here we wanted to investigate potential compensatory transcriptional response of the DNA glycosylase family to reduced levels of hMTH1 or hMYH protein levels. We speculated that *MTH1* and *MYH* double KD would lead to increased genomic oxidized lesions, and this may lead to compensational changes in expression of BER enzymes. To investigate this we performed qRT–PCR following single or double KD, and measured the expression level of all known human DNA glycosylases. *MTH1* and *MYH* double KD led to decreased *NEIL1* expression level to ∼30% in both hairpin sets (Figures 5a and b). No considerable changes were observed in the expression of other DNA glycosylases (Figure 5a). These results implicate NEIL1 in the cellular response to excess genomic oxidized lesions, caused here by *MTH1* and *MYH* double KD.

## Discussion

It has been suggested that processing of mis-incorporated 8-oxodG opposite dA is mainly mediated by post-replicative MMR in mammalian cells.^13^ In addition, hMYH activity has been shown to be required for removal of dA from the nascent strand when mispaired with 8-oxodG,^14^ and there is also cumulative evidence suggesting hMYH has physical interactions as well as functional cooperation with MMR.^14, 24^ Using data base mining, we observed *MYH* and *MTH1* expression levels are particularly high in T-ALL (Supplementary Figure S2). It has been reported that primary cells obtained from adult T-ALL patients are MMR inactive.^21, 25, 26^ Hence, this may reflect that hMYH and hMTH1 proteins are particularly important in the absence of MMR. Moreover, the majority of established T-ALL cell lines appeared to contain defects in MMR.^20, 27^ Therefore, we used MMR-defective T-ALL Jurkat A3 cells to study the interplay between hMYH and hMTH1. Supporting the involvement of MMR, hMTH1 overexpression has been reported to significantly decrease spontaneous mutation rate in MMR-defective cells, indicating an oxidized dNTP pool critically contributes to their genomic instability.^28^

Using two separate inducible vectors, we succeeded in knocking down *MTH1* and *MYH* separately or simultaneously (Figures 1b and c). *MTH1* suppression via *MTH1*-shRNA2 caused *MYH* upregulation, whereas the other hairpin, *MTH1*-shRNA1, showed a downregulation at 96 h that was also reported earlier.^8^ The upregulation of hMYH protein level in *MTH1*-shRNA2-expressing cells was confirmed using western blotting (Figure 1e). The varying response may be a reflection of the intricate relationship likely existing between the hMTH1 and hMYH proteins.

We found that simultaneous *MTH1* and *MYH* suppression increased the sub-G1 population significantly using both shRNA sets, showing that the two proteins collaborate for survival in T-ALL Jurkat A3 line. Interestingly, ongoing apoptosis, measured by Annexin V and cleaved caspase 3, at the 96 h time-point was different between the two treatments (Figure 3), likely a reflection of the different degrees of knockdown and timing of cell death between the conditions used. However, the specific role of hMYH in promoting survival was established by overexpression of the γ3-hMYH into *MTH1*-shRNA-containing cells that decreased overall cell death (Figure 4). These results support the hypothesis that hMYH and hMTH1 cooperate to avoid the accumulation of genomic DNA damage and consequent cell death.

To understand how *MYH* and/ or *MTH1* suppression can modulate the DNA repair pathways we analyzed the expression levels of human DNA glycosylases after 96 h of Dox treatment. Interestingly, we found that *NEIL1* expression was significantly reduced when both enzymes were depleted (Figure 5). Hydantoin lesions arising from oxidation of 8-oxodG are known substrates for NEIL1.^29^ In addition, NEIL1 exhibited pre-replicative repair of oxidized lesions preventing the progression of the nascent strands containing NEIL1 substrates. Accordingly, hyperactivation of NEIL1 has been reported to cause replication fork stalling.^30^ Based on this, we speculate that cells, with potential increased 8-oxodG levels caused by hMTH1 or hMYH loss, may suppress *NEIL1* expression to avoid incised hydantoin lesions (converted to DNA single-strand breaks) to cause replication fork collapse and DNA double-strand breaks. A speculative hypothesis, still to be tested, could be that the hydantoin lesion is less toxic and ‘better' for survival than conversion to potentially toxic BER intermediates.

In conclusion, this study provides evidence for the functional collaboration between hMYH and hMTH1 to maintain survival in cancer cells with MMR defective backgrounds, in particular T-ALL Jurkat A3 cells.

## Materials and methods

### Molecular cloning and DNA constructs

The shRNA plasmids were generated by cloning oligonucleotide from TRC library (Supplementary Table S1) into an inducible shRNA vector system, created through modifying pRSITEP-U6Tet-(sh)-EF1-TetRep-2A-Puro plasmid (Cellecta, Inc., Mountain View, CA, USA) by replacing the TetRep-2A-Puromycin site with either TetRep-P2A-Puro-P2A-RFP670 or TetRep-P2A-Puro-P2A-GFP. The vector contains a 1.2 kb stuffer sequence that was double–digested by FastDigest *Bsh*TI and *Eco*RI (FD1464 and FD0274; Thermo Fisher, Waltham, MA, USA) for 1 h. The 8 kb *Bsh*TI/*Eco*RI band was extracted from 1% agarose gel using Wizard SV Gel and PCR Clean-Up System (A9280; Promega, Madison, WI, USA). Separate forward and reverse oligos (custom DNA oligo service; Sigma) were annealed in the annealing buffer (10 mM Tris-HCl, pH 7.5, 0.1 M NaCl and 1 mM EDTA) using a thermal cycler (C1000; Bio-Rad), incubating for 5 min at 95 °C and then cooling gradually to 20 °C, and 1 μl of annealed oligo pairs was ligated into 50–100 ng of the digested vector using T4 DNA ligase (EL0014; Thermo Fisher) and T4 Polynucleotide Kinase (EK0031; Thermo Fisher), incubated overnight at 37 °C. Transformation was conducted using One Shot Stbl3 Chemically Competent Cells (C737303; Thermo Fisher) according to the manufacturer's protocol. Next, at least three colonies were picked for colony PCR using DreamTaq Green PCR Master Mix (K1081; Thermo Fisher). The primers used for this reaction were U6-tet-F: 5′-GGA CTA TCA TAT GCT TAC CGT AAC-3′ and U6-tet-R: 5′-TGG ATG AAT ACT GCC ATT TGT CTC-3′. Colonies lacking the stuffer were sent for sequencing (à la Carte Sequencing Service; Eurofins Genomics, Ebersberg, Germany). After sequencing validation of the constructs, a GFP reporter from pRSITEP-V2-GFP vector replaced the RFP670 cassette using double-digest reactions with FastDigest *Sal*I and FastDigest *Xba*I (FD0644 and FD0684; Thermo Fisher).

The γ3-hMYH overexpression plasmid was generated by PCR amplification of *MYH* cDNA (GenBank accession number AF527839.1) using the following primers: 5′-TAT AGT CGA CAT GAG GAA GCC ACG-3′ and 5′-TAT AGC GGC CGC TCA CTG GGC TGC ACT G-3′. Double digestion reactions were carried out with FastDigest *Sal*I and *Not*I (FD0644 and FD0593; Thermo Fisher) for 1 h. The PCR product was then ligated into pENTR1A no ccDB (17398; Addgene, Cambridge, MA, USA) entry vector using T4 DNA ligase (EL0014; Thermo Fisher). The entry vectors carrying γ3-hMYH insert were verified by sequencing (Eurofins Genomics). Next, the insert from entry vector was transferred to pINDUCER20 (44012; Addgene) destination vector using Gateway LR Clonase II (11791100; Thermo Fisher). Finally, the constructs were validated by colony PCR using DreamTaq Green PCR Master Mix (K1081; Thermo Fisher).

### Establishment of stable cell lines

Production of lentiviruses was performed following the protocol by Genetic Perturbation Platform, Broad Institute (http://www.broad.mit.edu/genome_bio/trc/rnai.html). Briefly, HEK-293T packaging cells were transfected by a shRNA vector together with packaging plasmids (12260 and 12259; Addgene) in 96-well plates. Virions were collected at 36 and 60 h post transfection and directly 25 μl of supernatant was used to transduce T-cell ALL cells in 96-well plates. Next day, cells were transferred into 24-well plates containing media with 0.5–1 μg/ml puromycin (A1113803; Thermo Fisher), selected for 6 days. Afterwards, top 15% of GFP–RFP670-positive cells were sorted in SciLifeLab FACS facility using BD Influx Cell Sorter (BD Biosciences, San Jose, CA, USA). For the cells transduced with only one shRNA plasmid, top 10% of RFP670-positive cells were sorted. Cells transduced with pINDUCER20 plasmids were selected for 12 days using 750 μg/ml G418 (G8168; Sigma, Darmstadt, Germany).

### Cell culture and treatments

T-cell ALL Jurkat A3 cell line was obtained from ATCC (Manassas, VA, USA) and grown in RPMI media (61870044; Thermo Fisher) with 10% fetal bovine serum (16140071; Thermo Fisher). Cells were cultured in T-75 flasks, treated with 250 ng/ml doxycycline hyclate (D9891; Sigma) and samples were collected at 96 h post-doxycycline treatment for downstream applications such as qRT–PCR, western blotting, flow cytometry and so on.

### qRT–PCR analysis

Total RNA was extracted from cells using Direct-zol RNA MiniPrep Kit (R2053; Zymo Research, Irvine, CA, USA). RNA (500 ng) was used to synthesize cDNA using iScript cDNA Synthesis Kit (1708891; Bio-Rad, Hercules, CA, USA). qRT–PCR reactions were carried out using primers listed in Supplementary Table S2 and iTaq Universal SYBR Green Supermix (1725124; Bio-Rad) on a CFX96 Touch Real-Time PCR Detection System (Bio-Rad). Analysis of data was performed using CFX Manager Software (Bio-Rad). The qRT–PCR experiments were carried out independently at least three times in technical triplicate.

### Western blotting analysis

Cells were washed once with cold phosphate-buffered saline (PBS) and then resuspended in a lysis buffer (50 mM Tris-Cl, pH 7.5, 150 mM NaCl, Protease Inhibitor Cocktail (04693116001; Roche, Basel, Switzerland), Halt Phosphatase Inhibitor Cocktail (78420; Thermo Fisher)). Cell lysis was prepared by three repeated thaw–freeze cycles at 37 °C and −80 °C. Pierce BCA Protein Assay Kit (34095; Thermo Fisher) was used to determine protein concentration in the samples. At least 12 μg of total lysate was loaded on the SDS–polyacrylamide gel electrophoresis. Membranes were blocked with PBS plus 0.05% (v/v) Tween-20 containing either 3% bovine serum albumin or 5% skimmed milk powder for 1 h at room temperature, followed by overnight incubation with primary antibodies at 4 °C. Incubation with secondary antibodies was carried out at room temperature for 45 min. For horseradish peroxidase (HRP)-conjugated secondary antibodies, SuperSignal West Femto Maximum Sensitivity Substrate (34095; Thermo Fisher) was used. Images were acquired using Odyssey Fc imager (LI-COR, Biosciences, Lincoln, NE, USA) and analyzed by Image Studio Lite (LI-COR). Where indicated, membranes were stripped by Restore Western Blot Stripping Buffer (21059; Thermo Fisher). Primary antibodies used were: GAPDH (sc-25778; Santa Cruz, Dallas, TX, USA), hMTH1 (NB100-109; Novus Biologicals, Littleton, CO, USA), hMYH (H00004595-M01; Abnova, Taipei, Taiwan) and Active Caspase-3 (ab32042; Abcam, Cambridge, UK). The following were used as secondary antibodies: Anti-Rabbit IgG-HRP conjugate (711-035-152; Jackson Immuno Research, West Grove, PA, USA), Anti-Mouse IgG-HRP conjugate (715-035-150; Jackson Immuno Research), Anti-Rabbit IgG-IRDye 680RD (925-68073; LI-COR) and Anti-Mouse IgG-IRDye 800CW (925-32212; LI-COR). The western blotting was performed independently at least three times.

### Flow cytometry

For DNA content measurement, cells were washed with PBS and fixated in cold 70% ethanol overnight. After resuspension of cell pellets in PBS with 1% bovine serum albumin, samples were incubated in DNA staining solution (10 μg/ml DAPI (D9542; Sigma), 0.1 mg/ml RNase A (EN0531; Thermo Fisher) and 1% bovine serum albumin in PBS) for 20 min at room temperature, followed by flow cytometry analysis on a Navios Flow Cytometer (Beckman Coulter, Brea, CA, USA). Data were analyzed using Kaluza Flow Analysis Software (Beckman Coulter).

For apoptosis detection, cells were washed with PBS and resuspended in Annexin V Binding Buffer (556454; BD Biosciences) containing 1:100 diluted Annexin V, Alexa Fluor 555 conjugate (A35108; Thermo Fisher) and 10 μg/ml DAPI (D9542; Sigma), incubated for 20 min at room temperature in the dark before flow cytometry analysis. The flow cytometry experiments were performed independently at least three times in technical duplicate.

### Statistical analysis

Multiple comparison between groups was performed by analysis of variance test using Prism 6 (GraphPad Software, La Jolla, CA, USA). Data are presented as mean±s.e.m. and differences with values of *P*<0.05 were considered to be statistically significant.
