# Supplementary material for: hMYH and hMTH1 cooperate for survival in mismatch repair defective T-cell acute lymphoblastic leukemia
Source: Oncogenesis. 2016 Dec 5;5(12):e275–. doi: 10.1038/oncsis.2016.72 (PMC5177770; doi:10.1038/oncsis.2016.72)
Supplement: Supplementary Information [file oncsis201672x1.docx]

**Supplementary Information**

**hMYH and hMTH1 Cooperate for Survival in Mismatch Repair Defective T-cell Acute Lymphoblastic Leukemia**

**Saeed Eshtad^1^, Zahra Mavajian^1^, Sean G. Rudd^1^, Torkild Visnes^1^, Johan Boström^1^, Mikael Altun^1^, and Thomas Helleday^1^**

^1^Science for Life Laboratory, Division of Translational Medicine and Chemical Biology, Department of Medical Biochemistry and Biophysics, Karolinska Institutet, S-171 21 Stockholm, Sweden.

*Correspondence and requests for materials should be addressed to T.H. (email: thomas.helleday@scilifelab.se).

**Supplementary Figure S1: Three main pathways are involved in the repair of 8-oxodG.**

When dATP is mis-inserted opposite 8-oxodG (OG), hMYH removes dA from the nascent strand. MUTSα, a component of MMR, recognizes the 8-oxodG:A mismatches where dA is on the template strand. In this case, MUYH activity is proposed to be inhibited and MUTSα instead would remove the OG. By converting 8-oxodGTPs to 8-oxodGMPs, hMTH1 prevents the incorporation of free oxidized nucleotides into DNA. See text for details.

**Supplementary Figure S2: T-ALL cells lines showed high expression of *MYH* and *MTH1*.** The expression levels of *MYH* (A) and *MTH1* (B) in over 900 cell lines are shown in box plots. Data is extracted from Cancer Cell Line Encyclopedia (CCL) (<http://www.broadinstitute.org/ccle>) ([1](#_ENREF_1)).

| **Oligo Sequence** | **TRC No.** | **Forward/**  **Reverse** | **shRNA** |
| --- | --- | --- | --- |
| CCGGCCTAAGGTTAAGTCGCCCTCGCTCGAGCGAGGGCGACTTAACCTTAGGTTTTTG | Sarbassov et al.([2](#_ENREF_2)) | F | *NT* |
| AATTCAAAAACCTAAGGTTAAGTCGCCCTCGCTCGAGCGAGGGCGACTTAACCTTAGG | Sarbassov et al.([2](#_ENREF_2)) | R | *NT* |
| CCGGCCTGCTTCAGAAGAAGAAATTCTCGAGAATTTCTTCTTCTGAAGCAGGTTTTTG | TRCN0000288945 | F | *MTH1-1* |
| AATTCAAAAACCTGCTTCAGAAGAAGAAATTCTCGAGAATTTCTTCTTCTGAAGCAGG | TRCN0000288945 | R | *MTH1-1* |
| CCGGCCCGACGACAGCTACTGGTTTCTCGAGAAACCAGTAGCTGTCGTCGGGTTTTTG | TRCN0000288946 | F | *MTH1-2* |
| AATTCAAAAACCCGACGACAGCTACTGGTTTCTCGAGAAACCAGTAGCTGTCGTCGGG | TRCN0000288946 | R | *MTH1-2* |
| CCGGCCACTGTGATCAACTACTATACTCGAGTATAGTAGTTGATCACAGTGGTTTTTG | TRCN0000056604 | F | *MYH-1* |
| AATTCAAAAACCACTGTGATCAACTACTATACTCGAGTATAGTAGTTGATCACAGTGG | TRCN0000056604 | R | *MYH-1* |
| CCGGCAAGCTGACATATCAAGTATACTCGAGTATACTTGATATGTCAGCTTGTTTTTG | TRCN0000056603 | F | *MYH-2* |
| AATTCAAAAACAAGCTGACATATCAAGTATACTCGAGTATACTTGATATGTCAGCTTG | TRCN0000056603 | R | *MYH-2* |

**Supplementary Table S1.** Sequences of oligos used for cloning shRNAs

| **Reverse** | **Forward** | **Accession** | **Target** |  |
| --- | --- | --- | --- | --- |
| AATCAGTGCCGTGGTTCGTG | CCCGAAACGCCGAATATAATCC | NM_003194 | *TBP* | Reference Genes |
| ATCTGCATTGTCAAGTGACGA | CGGTGAACGCCGATGATTAT | NM_021009 | *UBC* |  |
| GGCTGGTCGAAGAACTCCAA | CCCATACCGCAGCATCTATTT | NM_002434 | *MPG* | DNA Glycosylases |
| TTGAACACTAAAGCAGAGCCC | CCCCACACCAAGTCTTCACC | NM_003362 | *UNG* |  |
| CGAGTCACGTAGTTGCGATG | GAGGAGCTTCGGCTCAATG | NM_001243791 | *SMUG1* |  |
| GCAGAAGCGATGGGTTCTTGTA | CCGTCACCTCTAGTGAGCG | NM_003925 | *MBD4* |  |
| TTCCACTGGTTGTTTTGGTTCT | TGAAGCTCCTAATATGGCAGTTG | NM_003211 | *TDG* |  |
| GGATGAGCCGAGGTCCAAAAG | ACTCCCACTTCCAAGAGGTG | NM_016820 | *OGG1* |  |
| CTGCATCCATCCGGTATAGTAGT | GCCTGCTAAGCTGGTACGAC | NM_001048174 | *MYH* |  |
| CTCATGGCACGGATGTTGAC | CGCGGAAAGCACAGAGACT | NM_002528 | *NTHL1* |  |
| GTCCACGAAACATAGGGCGAG | CCTACCGCATCTCAGCTTCAG | NM_001256552 | *NEIL1* |  |
| GCACTCAGGACTGAACCGA | CTGTCTGCTATACACTGCTGGA | NM_145043 | *NEIL2* |  |
| GACCACAATTAGGACGCTTGTAA | TGGATCAGAACGTATTGCCTGG | NM_018248 | *NEIL3* |  |
| GTGGAAACCAGTAGCTGTCGT | GCTCATGGACGTGCATGTCTT | NM_198953 | *NUDT1*  *(MTH1)* | Nudix Hydrolase |

**Supplementary Table S2.** List of the qRT-PCR primers used in this study

**References**

1. Barretina J, Caponigro G, Stransky N, Venkatesan K, Margolin AA, Kim S, et al. The Cancer Cell Line Encyclopedia enables predictive modelling of anticancer drug sensitivity. Nature. 2012 03/29/print;483(7391):603-307.

2. Sarbassov DD, Guertin DA, Ali SM, Sabatini DM. Phosphorylation and regulation of Akt/PKB by the rictor-mTOR complex. Science (New York, NY). 2005 Feb 18;307(5712):1098-101. PubMed PMID: 15718470. Epub 2005/02/19. eng.
