# Supplementary figures and images for: hMYH and hMTH1 cooperate for survival in mismatch repair defective T-cell acute lymphoblastic leukemia
Source: Oncogenesis. 2016 Dec 5;5(12):e275–. doi: 10.1038/oncsis.2016.72 (PMC5177770; doi:10.1038/oncsis.2016.72)

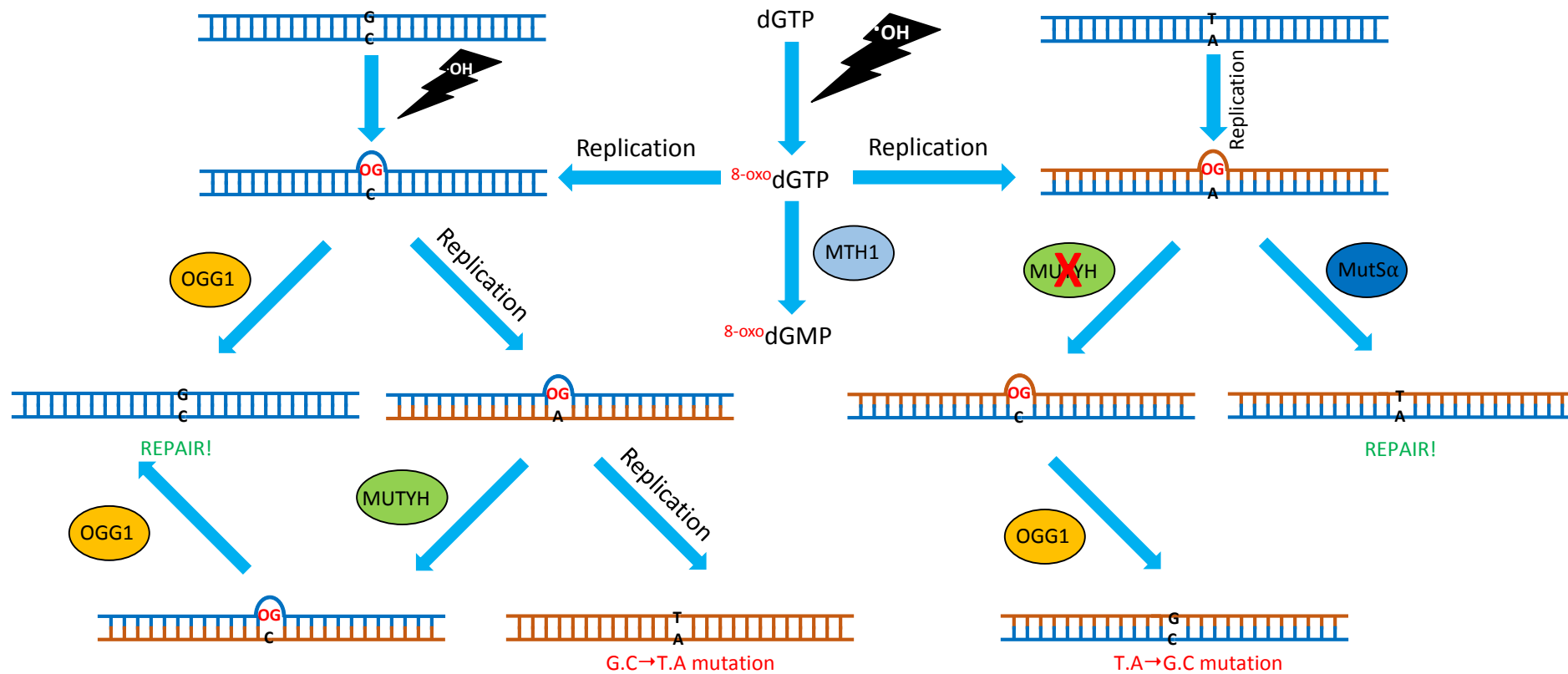

Supplement: Supplementary Figure 1 [file oncsis201672x2.pdf]

# MUTYH - Entrez ID: 4595

(A)

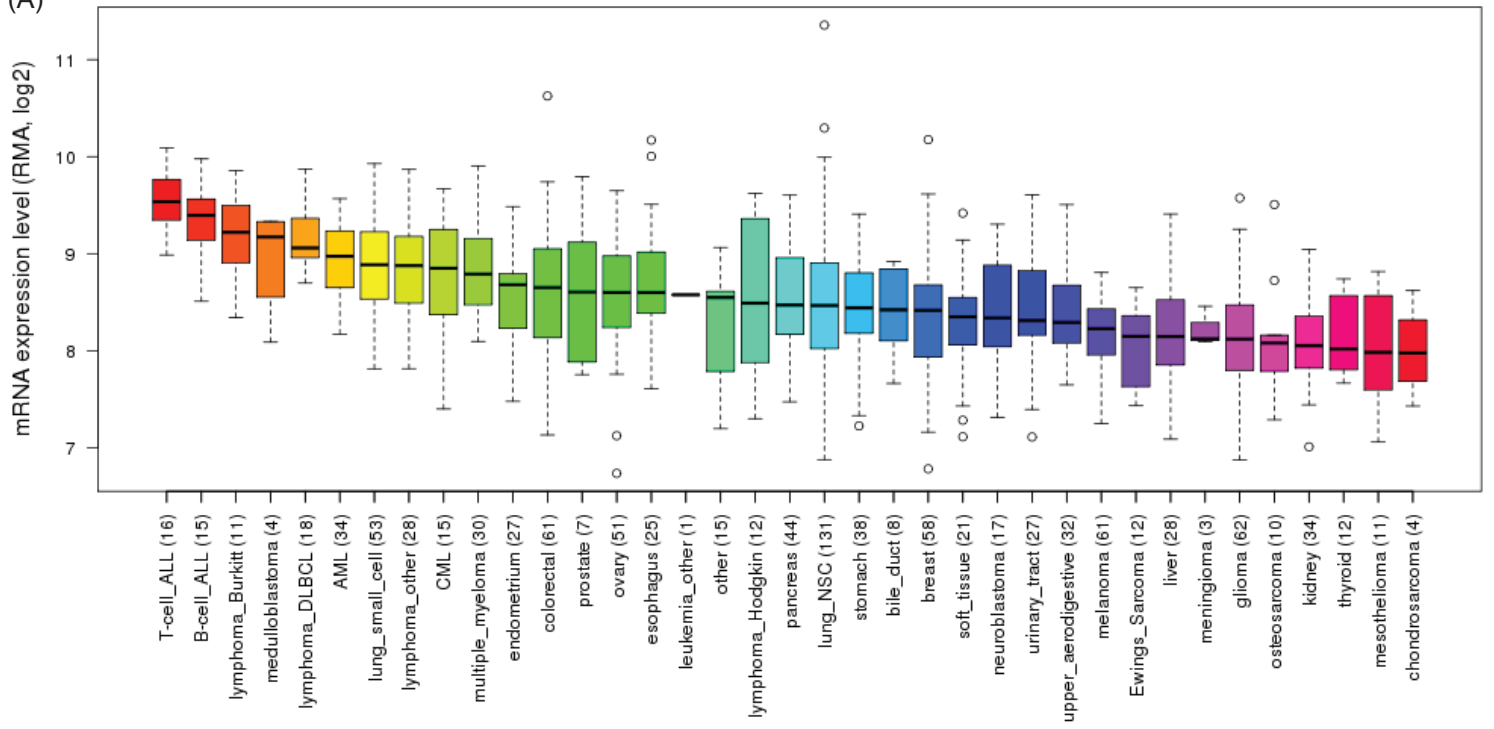

# NUDT1 - Entrez ID: 4521

(B)

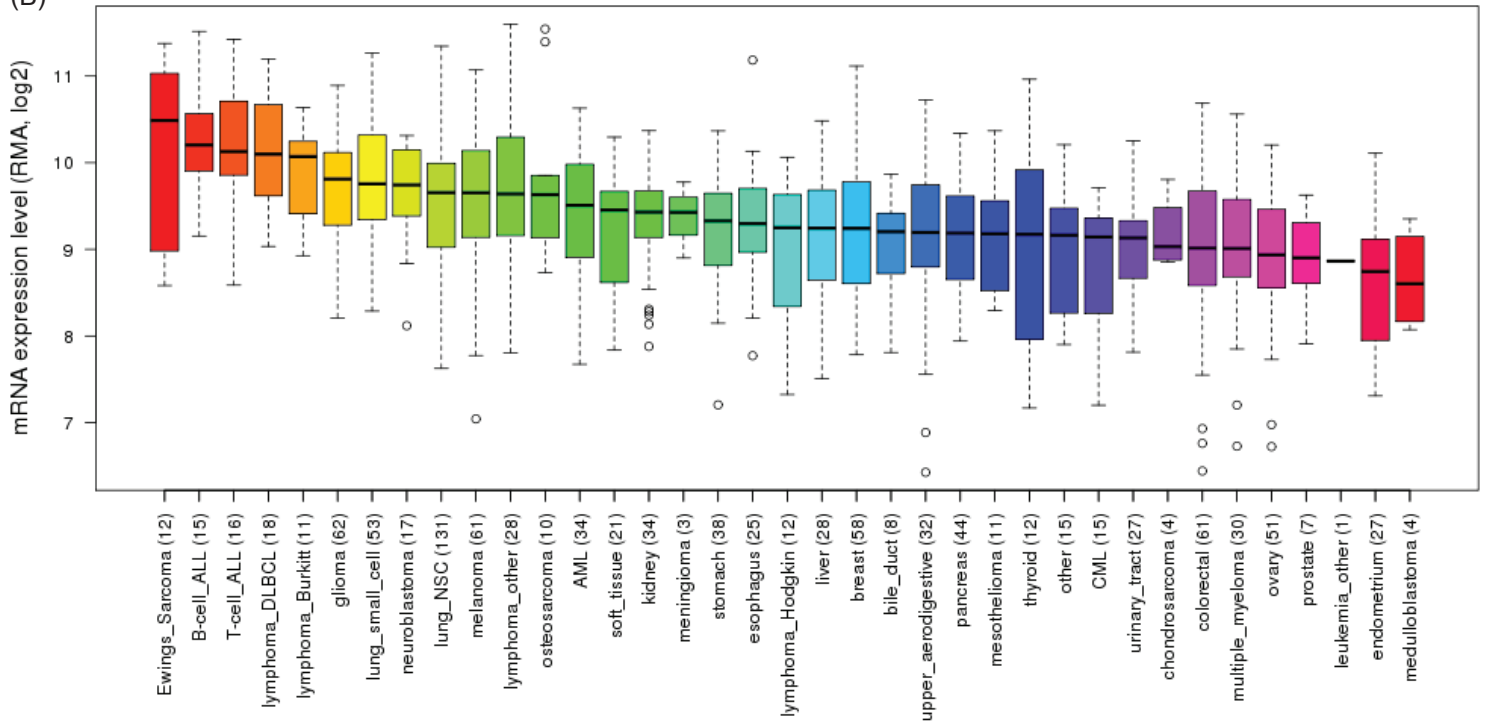

Supplement: Supplementary Figure 2 [file oncsis201672x3.pdf]
